# GaussianCpG: a Gaussian model for detection of CpG island in human genome sequences

**DOI:** 10.1186/s12864-017-3731-5

**Published:** 2017-05-24

**Authors:** Ning Yu, Xuan Guo, Alexander Zelikovsky, Yi Pan

**Affiliations:** 1 Department of Computer Science, Georgia State University, 25 Park Place, Atlanta, 30303 GA USA; 2 Department of Informatics, University of South Carolina Upstate, 800 University Way, Spartanburg, 29303 SC USA; 3 Computer Science and Mathematics Division, Oak Ridge National Laboratory, 1 Bethel Valley Rd, Oak Ridge, 37830 TN USA

**Keywords:** CpG island, Methylation, Gaussian model, Epigenetics, Energy distribution, CpG box

## Abstract

**Background:**

As crucial markers in identifying biological elements and processes in mammalian genomes, CpG islands (CGI) play important roles in DNA methylation, gene regulation, epigenetic inheritance, gene mutation, chromosome inactivation and nuclesome retention. The generally accepted criteria of CGI rely on: (a) %G+C content is ≥ 50%, (b) the ratio of the observed CpG content and the expected CpG content is ≥ 0.6, and (c) the general length of CGI is greater than 200 nucleotides. Most existing computational methods for the prediction of CpG island are programmed on these rules. However, many experimentally verified CpG islands deviate from these artificial criteria. Experiments indicate that in many cases %G+C is < 50%, CpG _*obs*_/CpG _*exp*_ varies, and the length of CGI ranges from eight nucleotides to a few thousand of nucleotides. It implies that CGI detection is not just a straightly statistical task and some unrevealed rules probably are hidden.

**Results:**

A novel Gaussian model, GaussianCpG, is developed for detection of CpG islands on human genome. We analyze the energy distribution over genomic primary structure for each CpG site and adopt the parameters from statistics of Human genome. The evaluation results show that the new model can predict CpG islands efficiently by balancing both sensitivity and specificity over known human CGI data sets. Compared with other models, GaussianCpG can achieve better performance in CGI detection.

**Conclusions:**

Our Gaussian model aims to simplify the complex interaction between nucleotides. The model is computed not by the linear statistical method but by the Gaussian energy distribution and accumulation. The parameters of Gaussian function are not arbitrarily designated but deliberately chosen by optimizing the biological statistics. By using the pseudopotential analysis on CpG islands, the novel model is validated on both the real and artificial data sets.

## Background

DNA genomes are punctuated by CpG islands where high profiles of CpG sites are densely contained in some genome regions. However, CpG contents in the entire human DNA genome are generally suppressed to only around 1% comparing with other combinations [1]. Scientists find that it is in CpG islands where many biological processes occur closely related with high density of CpG contents [2]. In vertebrate, DNA methylation usually occurs in CpG islands and adds an additional methyl to cytosine such that the gene silencing may be caused by the additional methyl. This subtle process can further give rise to gene regulatory differentiation and various epigenetic issues. However, conventional bisulfite modification-based methods to determine CpG islands and methylation regions are time-consuming [3]. Although new sequencing techniques are developed for whole genome assays, it is reported to be too costly [4]. Thus, computational methods for detection of CpG islands are fundamental and effective for many biological studies [5].

The first article about the computational prediction of CpG islands for vertebrate genome was published in [6], which proposed CpG island (CGI) problems and gave the definition of CGI that has been widely accepted by the later research. A milestone article [7] further constrained the CGIs within only gene promoters and excludes *Alu* repeat regions. However, recent studies have revealed that CGIs are not only in the area of gene promoters but also contained in the regions of both coding and non-coding [4, 8].

The computational methods for the detection of CpG island can be primarily classified into four categories in terms of their main algorithms. The first type is window-based methods [7, 9, 10] that use a scrolling window to scan through the genome and detect CGIs by these established statistical criteria. A canonical algorithm in [7] shifts a size-adjustable window for 1 *nt* each time to calculate the %G+C content and CpG _*obs*_/CpG _*exp*_ within the window until encountering the satisfied CpG island. Subsequently it shifts to next adjacent window and calculates it again until the window does not satisfy the criteria. At that time, it shifts back each *nt* until finding the last satisfied boundary window. This algorithm is widely used because it strictly follows the statistical criteria. Obviously, one of obvious drawbacks of this method primarily is that the window size determines the success of prediction. That is, the larger window increases the predictive granularity and lags the computing speed while the smaller window decreases the computing complexity and increases the probability of omitting a potential CGI. Another drawback is that it probably is too sensitive to predict a whole CGI where a CpG island can be divided into many trivial segments.

The second type is Hidden-Markov-Model-based (HMM) methods [1, 3, 11, 12]. These methods use the statistical transition model to compute transitive probability within CpG island and between CGIs. The transition probability between any two adjacent nucleotides are obtained in the training phase for CGI regions and non-CGI regions respectively. The probability of CG pair in CpG-rich region is much higher than that in non-CGI region. Thus, the log-likelihood ratio of the probabilities for CpG and non-CpG is calculated to reflect the difference between two regions for each possible sequence [12]. However, the variant patterns in CpG islands can easily add some implacable noises to prediction due to insufficient data training, resulting in that the performance of the HMM-based method is negatively affected. Moreover, it is computing-inefficient.

Third, density-based methods [13, 14] intuitively calculate the density of CpG sites, similar to statistical methods in window-based methods. The density of CpG island can be simply computed by taking into account the ratio of the number of CpG sites in the CpG island and the total length of the CpG island. Its basic idea is that it sets initial seeds to iteratively adjust the density variables and expand the CpG-rich regions. That is, initially it sets a low/loose threshold of density to find the approximate border of CpG islands and then use the high/strict thresholds to further detect where the borders are as long as the sequence within the borders meets the density requirement. The main drawback of this method is that it relies so much on the thresholds of the density that represents the simply linear relation between the number of CpG sites and the length of CpG island while the ground truth of CpG distribution in CpG islands probably cannot be delineated by the linear model.

The fourth is the distance-/length- based method [15] that clusters data by the distance between CpG sites and provides a fast way to predict CpG islands. Compared with other methods, this method studies the sequence property of primary structure between any two adjacent CpG sites, which provides a new perspective to understand the phenomena of CpG island. However, this method is criticized that it mainly depends on the composition of the sequence, resulting in different outputs for a same CGI in different contexts, and low predictive sensitivity with trivial results [13].

The aforementioned methods cannot pursue both the sensitivity and the specificity simultaneously. Either they can have high sensitivity with low specificity, or high specificity can be attained with the loss of the sensitivity. It also implies that the original definition of CGI perhaps deviates from the ground truth [16].

Our proposed model aims to fit the niche of previous work by presuming that each CpG site has the potential energy [17] that satisfy the Gaussian energy distribution along its primary structure. To some extent, the term of energy can be replaced by the term of pseudopotential [18]. The Gaussian model is proposed to reflect and simplify the principles of microscopical interactions in the complex human genome. The model is computed not by the linear statistical method but by the Gassian filter. Moreover, the parameters of Gaussian function are not arbitrarily designated but deliberately chosen by optimizing the biological statistics. Thus, it results in that the proposed method shows the better performance over other existing methods in detecting CpG islands.

## Methods

### Assumptions

In order to simplify the microscopical interactions in the DNA genome and reflect the general principles of the complex system, we propose the Gaussian model based on the following assumptions: (a) Each CpG site preserves the potential energy and the CpG-rich regions where energy are highly aggregated have more potential opportunities for epigenetic events. (b) Each CpG island is regarded as an energy field where only the contained CpG sites can affect mutually. (c) The energy of each CpG site is closely related to its primary structure or secondary/ tertiary structures. However, due to the uncertainty of unknown secondary or tertiary structures, its primary structure is the main determinant. (d) Since we consider only the primary structure of CpG islands, the energy in a certain location is directly relevant to its neighboring CpG sites [17]. Namely, the energy of each CpG site is distributed across its nearby regions. (e) The energy at each nucleotide within the CpG island is the sum of energy distributed by nearby CpG sites. (f) Each CpG site has the same magnitude of potential energy.

### Notations

We assume that a DNA genome sequence *s* with the length of *n* *nt* have *m* CpG islands each of which is notated as *CGI*_*i*_, *i*∈{1,2,…,*m*}. In any *CGI*_*i*_, its length is *l*_*i*_, in which *k* CpG sites lay on. At any CpG site *cpg*_*ij*_, *j*∈{1,2,…,*k*}, we assume that it preserves the energy *E*. The energy is distributed to its nearby nucleotides, which satisfy Gaussian model function *g*(*x*) where *x* is the relative distance to the corresponding CpG site and its directions, + and −, represent 5’ end and 3’ end respectively. The accumulated energy for any nucleotide position *x* in *CGI*_*i*_(*x*∈{0,1,…,*l*_*i*_−1}) is denoted as *G*_*i*_(*x*), which is the sum of distributed energy *g*_*ij*_(*x*) at this location.

### Gaussian model

We assume that each CpG site meets the Gaussian model [17, 18] as shown in Eq. 1.

1 $$  g(x)=\frac{E}{{\sqrt{2\pi}\sigma}}e^{\frac{{-x^{2}}}{{\sigma^{2}}}},  $$

where *x* is the relative distance from this nucleotide to the CpG site, *E* is the energy constant each CpG site preserves and *σ* determines the smoothness of energy distribution. When *σ*→0, it converges to an impulse function. From this formula, we can compute that its energy is distributed smoothly when *σ* becomes large. Therefore, *σ* determines the curve of the distribution and further influences the predictive accuracy of this model.

Further, we can calculate the accumulated energy at any position *x* ^′^ in the *CGI*_*i*_ as Eq. 2. *x* ^′^ is the absolute location in the CpG island while *x* is the relative distance to CpG sites. *x* ^′^=*T*(*x*) and *x*=*T* ^−1^(*x* ^′^) represent the linear transformation between *x* and *x* ^′^.

2 $$  G_{i} (x') = \sum\limits_{j = 1}^{k} {g_{ij} (x')}= \sum\limits_{j = 1}^{k} {g_{ij} (T(x))},  $$

where *j*∈1,2,…,*k* and *k* is the number of CpG sites within this CpG island *CGI*_*i*_. The mean of pseudopotential energy in *CGI*_*i*_ can be expressed in Eq. 3.

3 $$  \hat{G}_{i} = \frac{1}{{l_{i}}}\sum\limits_{x = 0}^{l_{i} - 1} {G_{i} (T(x))} = \frac{1}{{l_{i}}}\sum\limits_{x = 0}^{l_{i} - 1}{\sum\limits_{j = 1}^{k} {g_{ij} (T(x))}}  $$

$\hat {G}_{i}$ is a measure to evaluate the energy in the candidate area: the higher energy it preserves, the more likely the region can be a real CpG island.

### Parameters

The scarcity of CpG sites in DNA genome determines that CpG sites can bring larger amount of information compared with other regions. From this aspect, the energy proposed in GaussianCpG somehow look similar to information energy. However, in GaussianCpG model, the energy of CpG sites are presumed to distribute to surrounding areas in an energy-rich CpG island. The adjacent CpG sites overlap their energy with each other and keep the energy saturated in the region. Obviously, the distances between adjacent CpG sites affect the strength of energy in CpG islands. Additionally, an important assumption is that the influence of CpG sites is only limited to its surrounding area and the far distant CpG sites can barely affect the current location as our model. Thus, before setting the parameters of Gaussian model, we need to cluster the CpG sites so that only nearby CpG sites are considered. That is, identifying the clustering threshold is indispensable prior to setting the GaussianCpG parameters.

**Fig. 1:**
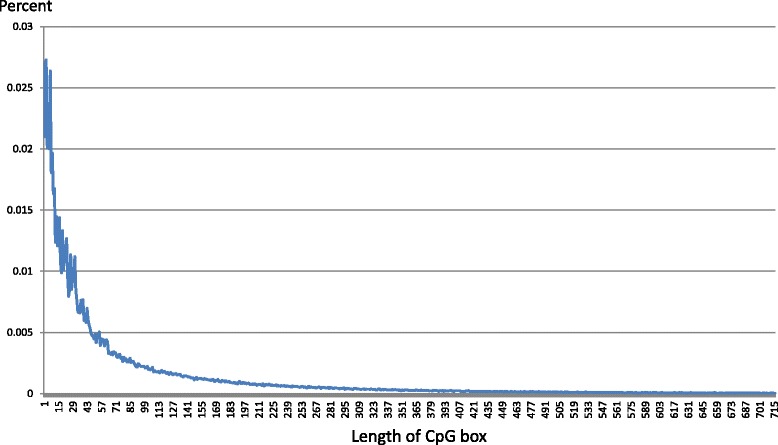
Distribution of CpG-box length. Distribution curve of CpG box in length for human genome

We use a new term, CpG box, to investigate the distribution of CpG distances and identify the clustering threshold. The CpG box is defined as the regions between two neighboring CpGs sites where nucleotides within the CpG dinucleotides are encapsulated likely in a box. We extract all CpG boxes and observe the distribution of all CpG-box lengths for human genome shown in Fig. 1. The distribution matches the kernel of exponential distribution. In [15] the curve was locally modeled as an approximate geometric distribution from around 20 *nt* to 100 *nt*, which did not reflect the ground truth of its distribution. In Eq. 4, *f*(*x*) is the distribution kernel and *x* is the length of CpG box, or say the distance between CpG sites.

4 $$  f(x) = \left\{{\begin{array}{cc} {\lambda e^{- \lambda x}} & {x \ge 0} \\ 0 & {x < 0} \\ \end{array}} \right.,  $$

where $\lambda = 1/\hat {x}$ and $\hat {x}$ is the mean length of CpG box. In terms of the exponential distribution in Eq. 4, the mean length is at $\hat {x} = 95$ while at the point of *x*=128 the third quarter of coverage is *ln*4/*λ*. By removing the under-represented value with large lengths (outlier), for example the length greater than 1k nt, we compromise and choose *x*=118 with about 73% coverage as the clustering threshold that eliminates the noises/outliers from extra large lengths and keeps the most reliable elements for further processing.

**Fig. 2:**
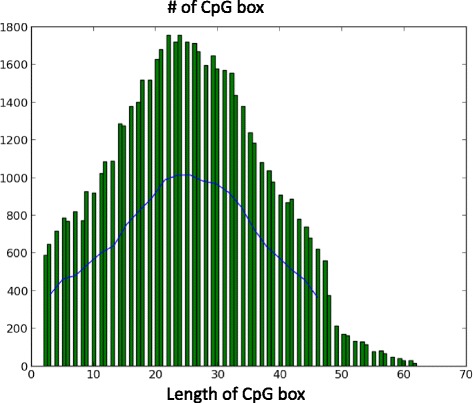
Gaussian kernel estimation. Distance distribution of CGI candidates in human chromosome 21 as an example and its Gaussian kernel density estimation (*blue solid line*)

**Fig. 3:**
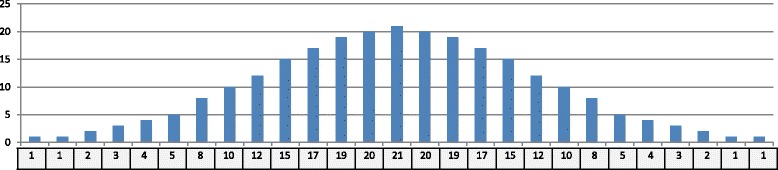
Discrete Gaussian filter. The upper chart shows the value for each location; the lower box is the discrete filter

By clustering the CpGs, we can minimize the range of potential CGIs. We extract CpG boxes from these CpG-rich regions and draw the distribution chart. It is found that the density estimation of this distribution fits Gaussian kernel as the blue solid line shown in Fig. 2 where human chromosome 21 is taken as an example. At the location of *x*=26 or *x*=27, the Gaussian kernel has the curve’s peak where the number of CpG-box length approaches the maximum. Hence, *x*=27 is chosen as the length of digital filter. In terms of Gaussian model in Eq. 1, the discrete Gaussian filter is created in Fig. 3.

### Implementation

**Fig. 4:**
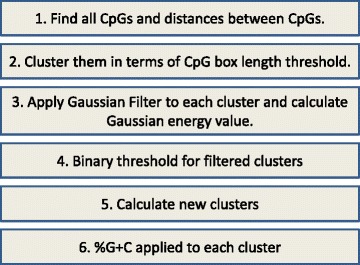
The main procedures of GaussianCpG

The main procedures of GaussianCpG are shown as Fig. 4: (1) Find all CpG sites for each human chromosome; (2) Cluster these CpGs in terms of the threshold of CpG-box length, namely the distance threshold between CpG sites; (3) Apply Gaussian filter to each cluster and calculate the magnitude of Gaussian potential energy; (4) Utilize a binary threshold to filter clusters; (5) Collect the filtered clusters; (6) Calculate %G+C for the remaining clusters and pick up those that meet the %G+C content. In the first step, all CpG sites and CpG boxes are extracted from genome as well as their properties, such as locations and lengths of CpG boxes. Note that the repeat regions are not included in this project following the conventional methods even if some literature [19] indeed stated that repeat area may involve more evolutionary force. Namely, we locate all CpGs’ positions first from annotated chromosome sequences and subsequently we divide a DNA sequence into sub-sequences by cutting at each CpG. Each sub-sequence that is also called CpG box has only two CpGs that are respectively located at its beginning and its end. Location information for CpG sites and CpG boxes are all stored. In the second step, using the statistical threshold *x*=118 we have acquired in statistics (described in the subsection of parameters), we cluster these CpGs into groups that may contain lots of CpG islands. The basic idea of clustering algorithm is to find all CpG boxes whose lengths are greater than threshold and then melt these CpG boxes from the sequence so that it is divided into segments. Subsequently, we apply Gaussian filter to scroll these clusters and calculate their energy value for each location. Segments can have the accumulated energy as well. After that, a binary filter is utilized to the computed loci in order to detect if these loci should be kept as CGI candidates, resulting in that new clusters are generated. That is, inside the large segment, it might be divided into sub-segments depending on the accumulated energy. The threshold we adopt here is 1.5 times of the average energy across the digital filter because of 2*δ* containing 95% energy in terms of Gaussian function. At the end, we count the percentage of %G+C content in these sub segments with the threshold of 40% and determine whether they are candidates.

For the computing complexity, the primary computing difficulty is the calculation of Gaussian filter applied to clustered CpG sites. To speed up the computation, we generate a matrix table that stores the computing intermediates to save the computational time. That is, for each location involved Gaussian filter computation, it takes the constant time for the calculation. Thus, its time complexity in Gaussian computation is *O*(*n*). For the rest computing tasks, extracting CpG sites takes *O*(*n*) and sorting the CpG distance takes *O*(*m* log*m*). *m* is the number of CpG sites and *n* is the sequence length, *m*<<*n*. Therefore, the time complexity of GaussianCpG is *O*(*n*). The program is implemented in Python and its libraries.

## Results

### Data set and Evaluation Metrics

In [15], in order to examine the capability of predicting those known CGIs for various methods, an artificial dataset was generated from the known data set, in which real CGIs were embedded into fake genome sequences. By detecting the real CGIs in those artificial sequences, the specificity and the sensitivity of the software can be easily validated since the details of true and false CGIs are exactly known. On the other hand, because the unknown/hidden CGIs may exist in the real genome data set, the validation in real data set is not so easy as that in artificial data. In the same vein as the literature, we generate an artificial data set to test the specificity of GaussianCpG. However, a little different from [15], the artificial data set are created by padding the gaps between known CpG islands using real human DNA sequences located at the regions between two CpG-rich areas instead of randomly padding nucleotides. The artificial data set contains 6,786 known CpG islands from the annotation database [20] with the nucleotide length of 6,854,696 *nt* and 6,786 non-CpG islands with the nucleotide length of 5,919,255 *nt*. The Lengths of CGIs vary from a hundred nucleotides to a few thousand of nucleotides.

In addition to artificial data set, in order to cross-validate our method, we use the real DNA genome data from UCSC annotation of Human Chromosome 21, which have been well researched as the benchmark of epigenetic data. It contains 348K annotated CGIs along with 46M DNA genome sequence.

Four mainstream software are examined in the performance evaluation of CpG-island prediction, including CpGPlot [10], CpGReport [10], CpGProd [9] and CpGCluster [15]. In the nucleotide level, the performance of each method is assessed by the observation of True Positive (TP), False Positive (FP), False Negative (FN), and True Negative (TN). Furthermore, the comprehensive assessments are defined and calculated, including sensitivity (Sn), specificity (Sp), accuracy (Acc), mean correlation coefficient (Mcc), positive predictive value (Ppv), performance coefficient (Pc) and F1 score.

### Experimental Results

For the general performance, Table 1 merely manifests the coverage rate of GaussianCpG for predicting those known CpG islands from the artificial data, where its average rate is 99.32%. Furthermore, Table 2 shows the comprehensive analysis for method comparison on artificial data set. The top one in sensitivity is CpGProd while its specificity is in the last rank, and the top one in specificity is CpGCluster while its sensitivity is near the worst. It means that those methods can hardly approach the point where both sensitivity and specificity are excellent. Whereas, the performance of GaussianCpG in both sensitivity and specificity are very near the top one, resulting in that its accuracy, predictive value, performance coefficient and the harmonic mean of sensitivity and precision are ranked as the top.

**Table 1:** Coverage rate of known human CGIs

Chr#	Known	Predicted	Coverage
Chr 1	546	541	99.08%
Chr 2	430	426	99.07%
Chr 3	319	319	100%
Chr 4	272	272	100%
Chr 5	359	356	99.16%
Chr 6	293	292	99.66%
Chr 7	304	298	98.03%
Chr 8	254	253	99.61%
Chr 9	359	356	99.16%
Chr 10	311	311	100%
Chr 11	346	346	100%
Chr 12	363	360	99.17%
Chr 13	200	200	100%
Chr 14	206	205	99.51%
Chr 15	150	150	100%
Chr 17	383	380	99.22%
Chr 18	43	43	100%
Chr 19	315	314	99.68%
Chr 20	259	257	99.23%
Chr 21	133	131	98.50%
Chr 22	215	214	99.53%
Chr X	253	250	98.81%
Chr Y	5	5	100%

Known CGIs: 6786, & predicted: 6740, & avg. coverage rate: 99.32%

**Table 2:** Comparison in artificial data set

^a^Method:	I	II	III	IV	V
T	6854696	6854696	6854696	6854696	6854696
TP	2101562	3603662	5489738	2531549	5036243
FN	4753134	3251034	1364958	4323147	1818453
F	5919255	5919255	5919255	5919255	5919255
FP	20437	220957	1085303	9319	46906
TN	5898818	5698298	4833952	5909936	5872349
^b^Method:	I	II	III	IV	V
Sn	30.66%	52.57%	**80.09%**	36.93%	73.47%
Sp	99.65%	96.27%	81.66%	**99.84%**	99.21%
Acc	62.63%	72.82%	80.82%	66.08%	**85.40%**
Mcc	99.04%	94.22%	83.49%	**99.63%**	99.08%
Ppv	30.57%	50.93%	69.14%	36.88%	**72.97%**
Pc	40.61%	53.18%	61.61%	45.94%	**74.04%**
F1	46.82%	67.49%	81.75%	53.89%	**84.37%**

I:CpGPlot, II:CpGReport, III:CpGProd, IV:CpGCluster, V:GaussianCpG

For Panel ^a^: The unit of measurement is necleotide

True, T: the length of known CpG islands

False, F: the length of non-CpG islands

True positive, TP: the length of predicted known CGIs

False positive, FP: the length of predicted CGIs not in known CGIs

False negative, FN: the length of not predicted known CGIs

True negative, TN: the length of predicted non-CGIs

For Panel ^b^:

Sensitivity, *Sn*=*TP*/(*TP*+*FN*)

Specificity, *Sp*=*TN*/(*TN*+*FP*)

Accuracy, *Acc*=(*TP*+*TN*)/(*TP*+*FP*+*FN*+*TN*)

Mean correlation coefficient,

${\qquad }{Mcc}={\textstyle {{{TP}\times {TN} - {FN}\times {FP}} \over {\sqrt {({TP} + {FN}) \times ({TN} + {FP}) \times ({TP} + {FP}) \times ({TN} + {FN})} }}}$

Positive predictive value, *Ppv*=*TP*/(*TP*+*FP*)

Performance coefficient, *Pc*=*TP*/(*TP*+*FN*+*FP*)

F1 score, the harmonic mean of precision and sensitivity,

*F*1=2×*TP*/(2×*TP*+*FP*+*FN*)

For Panel ^a^&^b^: Default parameters for all software are set

Table 3 shows the results for real data set, similar to Table 2. The bold values are the top results over others in corresponding rows. One drawback for real-data benchmark is that the sequence may contain some real CpG islands that probably are not annotated and some undiscovered ground truth may be involved, it gives rise to the increased False Positive for all programs. Thus, controlling the False Positive is the key to compete. This comparison shows the comprehensive ability of GaussianCpG in specificity, accuracy, mean correlationcoefficient, positive predictive value, performance coefficient and F1 score. The only inferior metric is in the sensitivity where GaussianCpG is listed in the medium level, close to CpGCluster but better than CpGPlot.

**Table 3:** Comparison in real data set

^a^Method:	I	II	III	IV	V
T	348930	348930	348930	348930	348930
TP	255732	348546	333015	300315	292732
FN	93198	384	15915	48615	56198
F	46361053	46361053	46361053	46361053	46361053
FP	397423	1680731	1034353	583460	363493
TN	46124740	44417698	45331765	45923959	46075369
^b^Method:	I	II	III	IV	V
Sn	73.29%	**99.88%**	95.43%	86.06%	83.89%
Sp	99.14%	96.35%	97.76%	98.74%	**99.21%**
Acc	98.95%	96.38%	97.75%	98.65%	**99.10%**
Mcc	53.11%	40.65%	47.61%	53.60%	**60.80%**
Ppv	39.15%	17.17%	24.35%	33.98%	**44.60%**
Pc	34.26%	17.17%	24.07%	32.20%	**41.08%**
F1	51.03%	29.31%	38.80%	48.72%	**58.24%**

I:CpGPlot, II:CpGReport, III:CpGProd, IV:CpGCluster, V:GaussianCpG

For Panel ^a^&^b^: The setting and metrics are same as those in Table 2

Note that the parameters of methods used in the comparison are from the default setting of their systems while GaussianCpG adopts the default parameters described in aforementioned sections.

From the validation experiments, we can observe that the GaussianCpG model is a comprehensive method that can balance both sensitivity and specificity and manifest the excellent performance in predicting CpG islands in human DNA genome. The main reasons that GaussianCpG can achieve better performance than other models probably lie on three factors: (1) GaussianCpG is designed on the fine-grained statistic analysis throughout the whole human genome rather than coarse-grained threshold-based criteria, which drives the generation of the Gaussian model. (2) The established Gaussian model probably coincides with some statistics of hidden bio-chemical patterns that are still not discovered and unknown so far. (3) GaussianCpG measures the structural properties of CpG box such as distance and energy distribution, rather than arbitrary thresholds, that are probably related to some undiscovered DNA structures.

As for the running time, Gaussian filter takes a linear time to filter all CpGs throughout sequences. Namely, there are a constant number of calculations for each base position, which we have discussed in Section 3. For large scale human chromosomes (totally 3 GBytes), a sequential program, written in python running on an Intel i7 CPU with 8G RAM, takes less than 20 minutes for the entire analysis, and hence is prompt enough to handle large-scale genome input.

## Discussion

A novel Gaussian model, GaussianCpG, is developed for detection of CpG islands on human genome. We analyze the energy distribution over genomic primary structure for each CpG site and adopt the parameters from statistics of Human genome. It exposes that GaussianCpG is a species-specific method. GaussianCpG currently is only designed for human genome. That is, the parameters should be different between species such as mouse and human.

Therefore, some work are remained to the future. First of all, it needs to be further tested on other species for its generality and applicability, especially on vertebrates, although GaussianCpG initially was designed for human genome. It is because CpG clustering is often regarded as a species-specific issue [21]. Second, the pattern of CpG structure is still undiscovered. Statistical data can only give an observation to the phenomena but cannot give the reason. In [22], statistics were given while underlying bio-chemical or bio-physical analysis were needed. From this perspective, energy analysis based on bio-chemical or bio-physical data [23] probably is a right direction to unveil the CpG sparsity that may further determine the structure of CpG island. That is, integrating statistics and molecule chemistry/dynamics might be a good combination to reveal those non-conserved patterns and hidden rules.

## Conclusion

In summary, GaussianCpG is a novel Gaussian model applied to human genome for epigenetic studies. The design of GaussianCpG simplifies the interaction of molecules and delineates the substantial procedure that may affect epigenetic issues in the complex human DNA genome. The comparative results show that GaussianCpG can provide a reliable way for prediction of CpG island and benefit the research on methylation and epigenetics. In addition, GaussianCpG examines the CpG islands from an unique perspective different from other existing methods. It analyzes the statistics of CpG islands and constructs an elaborate Gaussian filter. By using the pseudopotential analysis on CpG islands, the novel GaussianCpG model can promote the performance on the real and artificial data sets and it is validated as a more effective model for computationally detecting the CpG islands on Human genome sequences.
